# Supplementary material for: From sequence to enzyme mechanism using multi-label machine learning
Source: BMC Bioinformatics. 2014 May 19;15:150. doi: 10.1186/1471-2105-15-150 (PMC4229970; doi:10.1186/1471-2105-15-150)
Supplement: Additional file 2 — Java code of ml2db. Additional file ml2db_code.tar.gz contains the Java source code to run the multi-label machine learning experiments and save the results to database. The code’s Javadoc is included. [file 1471-2105-15-150-S2.zip › additional file 2/ml2db/ml2db/doc/mulan/data/class-use/LabelsPair.html]

Uses of Class mulan.data.LabelsPair


JavaScript is disabled on your browser.


- Overview
- Package
- Class
- Use
- Tree
- Deprecated
- Index
- Help

- Prev
- Next

- Frames
- No Frames

- All Classes

## Uses of Class mulan.data.LabelsPair

- Packages that use LabelsPair

  | Package | Description |
  | --- | --- |
  |  |  |
  | --- | --- |
  | mulan.data |  |
- - ### Uses of LabelsPair in mulan.data

    Methods in mulan.data that return LabelsPair

    | Modifier and Type | Method and Description |
    | --- | --- |
    |  |  |
    | --- | --- |
    | `LabelsPair[]` | LabelPairsDependenceIdentifier.`calculateDependence(MultiLabelInstances mlInstances)` Calculates dependence level between each pair of labels in the given multilabel data set |
    | `LabelsPair[]` | UnconditionalChiSquareIdentifier.`calculateDependence(MultiLabelInstances mlInstances)` Calculates Chi Square values for each pair of labels. |
    | `LabelsPair[]` | ConditionalDependenceIdentifier.`calculateDependence(MultiLabelInstances mlInstances)` Calculates t-statistic value for each pair of labels. |

- Overview
- Package
- Class
- Use
- Tree
- Deprecated
- Index
- Help

- Prev
- Next

- Frames
- No Frames

- All Classes
